# Distinct patterns of dentate gyrus cell activation distinguish physiologic from aberrant stimuli

**DOI:** 10.1371/journal.pone.0232241

**Published:** 2020-05-14

**Authors:** Jason C. You, Kavitha Muralidharan, Chia-Hsuan Fu, Jin Park, Umberto Tosi, Xiaohong Zhang, Jeannie Chin

**Affiliations:** 1 Department of Neuroscience and Farber Institute for Neurosciences, Thomas Jefferson University, Philadelphia, Pennsylvania, United States of America; 2 Memory & Brain Research Center, Department of Neuroscience, Baylor College of Medicine, Houston, Texas, United States of America; University of Modena and Reggio Emilia, ITALY

## Abstract

Under physiologic conditions, the dentate gyrus (DG) exhibits exceptionally low levels of activity compared to other brain regions. A sparse activation pattern is observed even when the DG is engaged to process new information; for example, only ~1–3% of neurons in the DG granule cell layer (GCL) are activated after placing animals in a novel, enriched environment. Moreover, such physiologic stimulation of GCL neurons recruits young granule cells more readily than older cells. This sparse pattern of cell activation has largely been attributed to intrinsic circuit properties of the DG, such as reduced threshold for activation in younger cells, and increased inhibition onto older cells. Given these intrinsic properties, we asked whether such activation of young granule cells was unique to physiologic stimulation, or could be elicited by general pharmacological activation of the hippocampus. We found that administration of kainic acid (KA) at a low dose (5 mg/kg) to wildtype C57BL/6 mice activated a similarly sparse number of cells in the GCL as physiologic DG stimulation by exposure to a novel, enriched environment. However, unlike physiologic stimulation, 5 mg/kg KA activated primarily old granule cells as well as GABAergic interneurons. This finding indicates that intrinsic circuit properties of the DG alone may not be sufficient to support the engagement of young granule cells, and suggest that other factors such as the specificity of the pattern of inputs, may be involved.

## Introduction

The dentate gyrus (DG) of the hippocampal formation plays a vital role in transforming spatial information into neuronal representations of memory. Consistent with its function, neuronal activity in the DG is tightly controlled, occurring in a sparse and selective pattern after physiologic stimulation [1–3]. The specificity of activation is widely attributed to two unique properties of the DG neural network: 1) strong local GABAergic inhibition, and 2) adult neurogenesis that adds new principal neurons (i.e. granule cells) to the granule cell layer (GCL) of the DG [4–6].

Previous studies have shown that during a critical period of granule cell maturation (6–8 weeks of age), young granule cells begin to form strong reciprocal connections with GABAergic interneurons that limit their excitability beyond 8 weeks of age [7–9]. Therefore, physiologic stimulation of the DG more readily activates young granule cells (<8 weeks old), which have not yet established these robust inhibitory connections [10–13]. Conversely, old granule cells (≥8 weeks old), which comprise the majority of cells in the GCL, are efficiently inhibited by GABAergic interneurons and remain largely silent when the DG receives input. Such “old” granule cells include granule cells that were born prenatally as well as those born postnatally but have developed and matured for at least 8 weeks. The combination of effects from network inhibition and the more ready engagement of young granule cells contribute to why only ~1–3% of neurons in the GCL are activated by exposure to physiologic stimuli that trigger new information coding and memory formation [11, 12, 14, 15]. The sparse activation of young granule cells in the GCL under physiologic conditions is thought to contribute to pattern separation, a DG-dependent function that allows similar but distinct memories to be distinguished from one another [13, 16, 17].

However, whether the sparse pattern of granule cell activation that favors young granule cells is achieved primarily by the presence of local circuit properties (e.g., time-delayed formation of inhibitory contacts onto newborn granule cells) or is influenced by other factors such as the specificity of input to the DG, is not clear. This question is important to address since the DG can be subject to a variety of physiologic and pharmacologic stimuli, often with downstream behavioral consequences [18–25].

To assess whether the etiology of DG stimulation impacts pattern of cellular activation, we compared the activation of cells in the DG granule cell layer by two different modes of stimulation. One mode was physiologic stimulation by exposure of mice to a novel, enriched environment; the other mode was pharmacological activation of the hippocampus by a low dose of kainic acid. We found that both modes of stimulation activated a similarly sparse number of cells in the dentate granule cell layer. However, although exploration of a novel, enriched environment engaged both young and older granule cells as expected, low dose kainic acid engaged only older granule cells and GABAergic interneurons. Our results are consistent with the hypothesis that factors in addition to local circuit and network properties are necessary for the engagement of younger dentate granule cells by physiologic stimulation.

## Materials and methods

### Animals

A total of 82 mice were used in this study, which consisted of male and female C57BL/6J mice from Jackson laboratory. The average age of mice in different experiments varied between 2–6 months of age, but mice within an experiment had dates of birth that were within several weeks of each other. Animals were housed on a 12:12 light:dark cycle. No specific methods of randomization were used, but animals were semi-randomly assigned to experimental groups based on birth order after balancing for age and sex. For brain harvesting, mice were anesthetized with isoflurane or intraperitoneal injection of a ketamine/xylazine overdose, and then transcardially perfused with ice-cold saline (0.9% NaCl). The brains were extracted and fixed in 4% paraformaldehyde for 48 hours at 4°C. All experimental procedures were approved by the Institutional Animal Care and Use Committees at Thomas Jefferson University and Baylor College of Medicine.

### Kainic acid (KA) seizures

Kainic acid monohydrate (Sigma-Aldrich, K0250) was dissolved in saline at a concentration of 2 mg/mL working solution and frozen in aliquots. On the days of KA administration, fresh aliquots were thawed at room temperature and administered via intraperitoneal injection at a final dose of 5 mg/kg or 15 mg/kg based on animal weight. Equal volumes of saline were administered as control. Mice injected with KA were monitored for 2 hrs after injection to score behavior as follows: 0, normal behavior; 1, immobility; 2, generalized spasm, tremble, or twitch; 3, tail extension; 4, forelimb clonus; 5, generalized clonic activity with loss of posture; 6, bouncing or running seizures; 7, full tonic extension; 8, death. Mice were sacrificed either 2 hrs or 3 days after KA administration.

### EEG recordings

Mice were stereotaxically implanted with a six-electrode array headcap for EEG monitoring, as described in [26]. Briefly, teflon-coated silver wire (0.005 in diameter) attached to a 6-pin Delran pedestal (Plastics One) was wrapped around screws implanted bilaterally into the subdural space over frontal and temporal cortices (from Bregma:1.0 mm A-P, 1.5 mm M-L; -2.2 mm A-P, 2 mm M-L) along with a hippocampal depth electrode (-2.2 mm A-P, 2 mm M-L,1.8 mm from brain surface (DV)). Ground and reference electrodes were implanted directly behind Lambda on either side of the midline. Mice were allowed to recover for at least 4 days before recordings were conducted. EEG recordings were performed in the home cage of the mice for three continuous days after injection with 5 mg/kg KA, or for one day after injection with 15 mg/kg KA, using a Stellate Harmonie acquisition system (version 7.0a, Natus Medical, Pleasonton, CA) with a sampling rate of 2000 Hz for data acquisition. Native Stellate and Lab Chart Pro (AD Instruments Inc.) software were used for EEG signal processing and spike count analyses.

### BrdU administration

5-Bromo-2’-deoxyuridine, BrdU (Sigma, B5002), was dissolved in saline at a concentration of 10 mg/ml and stored at 4°C. On the days of BrdU administration, fresh aliquots were used to inject mice. Intraperitoneal injections of 100 mg/kg of BrdU were performed twelve hours apart, twice a day over the course of 5 days (total of 10 injections). Novel environment exposure or 5 mg/kg KA injections were performed 6 weeks later.

### Enriched environment paradigm

The enriched environment (EE) paradigm followed a published protocol [26] in which mice were placed in a novel cage containing a novel bedding type (Aspen wood shavings), a tray from a pipette box tip, a repeat pipetter tip wrapped in red tape, a pool of water (in a pipette box cover) with a floating 1-cm thick piece of banana, and a blue cap from a 50-mL conical vial. Mice were allowed to explore the novel, enriched environment for 2 hours while being video-recorded from above by a digital camcorder. Following the 2 hrs of EE exposure, mice were sacrificed immediately along with control mice that had remained in their home cages.

### Immunohistochemistry

Brains were cryoprotected in 30% sucrose in PBS, and coronal sections were cut with a freezing sliding microtome (Microm). Sections were distributed into ten subseries, each containing every tenth section throughout the rostral-caudal extent of the brain; each subseries therefore includes 8–10 sections that contain hippocampus. One full subseries of free-floating sections per mouse was used for each immunostain. After blocking nonspecific binding with 10% species-appropriate serum in PBS + 0.05% Triton X-100, sections were incubated overnight at 4°C with primary antibody diluted in PBS + 0.05% Triton X-100 including 3% serum. Primary antibodies included: rabbit anti-cFos (1:1,000, Calbiochem PC38 and 1:3,000, Millipore ABE457), mouse anti-calbindin (1:1,000, Swant 300), goat anti-doublecortin (1:100, Santa Cruz sc-8066), mouse anti-GAD67 (1:1,000, Millipore MAB5406), and rat anti-BrdU (1:1,000, Accurate Chemical, OBT0030G). Sections were then rinsed with PBS + 0.05% Triton X-100 before incubation with secondary antibodies for 2 hours at room temperature. Secondary antibodies included goat anti-rabbit FITC (1:200, Jackson Labs cat # 111-095-144), goat anti-mouse rhodamine (1:200, Jackson Labs cat # 115-295-166), donkey anti-goat Cy3 (1:200, Jackson Labs cat # 705-165-147), goat anti-rat Alexafluor 594 (1:500, ThermoFisher cat # A-11007) and goat anti-rabbit Alexafluor 488 (1:500, ThermoFisher cat # A-11034). For immunostaining of BrdU, sections were first treated with 2M HCl for 30 minutes at 37°C and 0.1M sodium tetraborate at room temperature before blockade of nonspecific binding. For all antibodies, after secondary antibody incubation, sections were rinsed in PBS + 0.05% Triton X-100 and in PBS. Stained sections were mounted on glass slides, and Prolong Gold with DAPI antifade mounting medium (ThermoFisher) was used to allow visualization of nuclei.

### Imaging and cell quantification

Fluorescent images were captured on a Zeiss AxioImager M2 epifluorescence microscope with a 10X objective, using MetaMorph software (Molecular Devices). For each mouse, images were taken of every section containing the dentate gyrus throughout its rostral-caudal extent. The same exposure settings were used to image all mice within each experiment. Cell counts were performed using ImageJ on 8 sections spanning the rostral-caudal extent of the dentate gyrus for each mouse. Immunoreactive cells within the dentate granule cell layer were counted in each section, and summed for each mouse. The person performing the quantification was blinded with respect to experimental condition of the mice.

### Statistics

Statistical analyses were performed using Prism 5 (GraphPad Software, San Diego, CA). Unless otherwise stated, results are represented as sample means ± standard errors of the mean. Differences between 5 mg/kg KA and 15 mg/kg KA with regard to seizures and spike counts were assessed using a Mann Whitney U test to compare total numbers of seizures or spikes, or two-way repeated measures ANOVA to compare occurrence of seizures/spikes over the recording period. For all other comparisons, differences between two independent sample means were assessed using a two-tailed, unpaired Student’s t-test. Differences among 3 or more sample means were assessed via one-way ANOVA when sample variances were equal and Brown-Forsythe ANOVA when sample variances were unequal, or a two-way ANOVA when there were two independent experimental variables involved. Subsequent post hoc analyses were applied when appropriate to detect focal differences between groups, and include Tukey’s Honestly Significant Difference, Fisher’s Least Significant Difference, and Dunnett T3 tests. For all cell count analyses, the experimenters were blinded to the treatment of each mouse.

## Results

### c-Fos expression in the dentate granule cell layer is increased to a similar extent by physiologic and pharmacological stimuli

**Fig 1:**
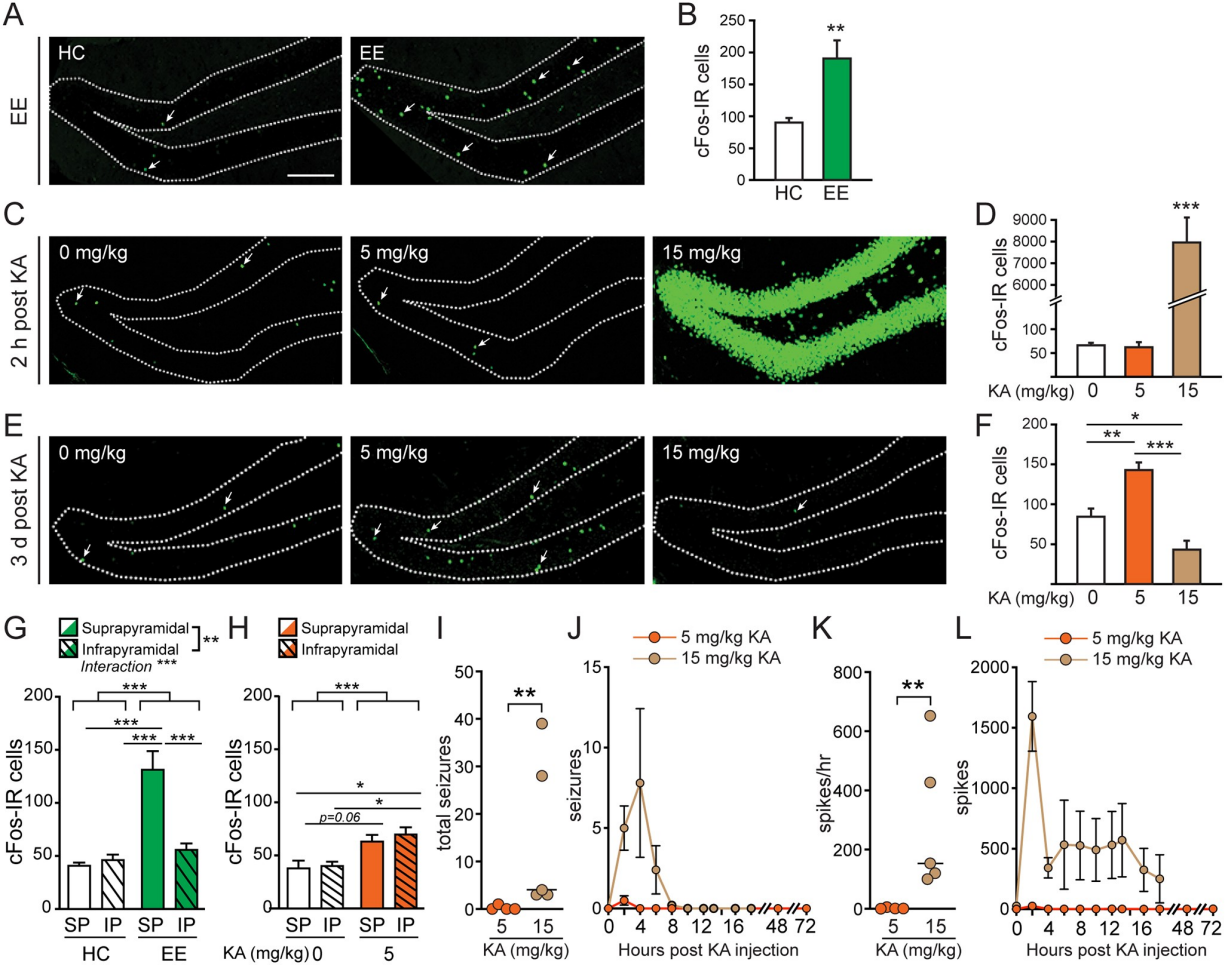
Low-dose KA (5 mg/kg) or exploration of a novel enriched environment (EE) increases GCL cFos expression in a similarly sparse manner. *A*, *B*: Images and quantification of cFos immunoreactive (IR) cells in the GCL after 2-hour exposure to EE (n = 8 per group; **p = 0.0043). *C*, *D*: Representative images and quantification of cFos IR cells in the GCL 2 hours after administration of 0, 5, or 15 mg/kg KA (n = 5 per group; ***p = 8x10^-6^). *E*, *F*: Images and quantification of cFos IR cells in the GCL 3 days after administration of 0, 5, or 15 mg/kg KA (n = 4 per group; 15 vs. 0 mg/kg, *p = 0.047; 5 vs. 0 mg/kg, **p = 0.0078; 15 vs. 5 mg/kg, ***p = 0.0002). *G*: Quantification of cFos IR cells in the suprapyramidal (SP) and infrapyramidal (IP) blades of the dentate gyrus after 2-hour exposure to EE. Two-way ANOVA found significant effects of treatment (p < 0.0001), blade (p = 0.0013), and an interaction (p = 0.0003; n = 8 per group). Posthoc tests revealed ***p<0.0001 for HC-SP vs EE-SP blades, EE-SP vs HC-IP blades, and EE-SP an EE-IP blades. *H*: Quantification of cFos IR cells in the SP and IP blades of the dentate gyrus 3 days after administration of 0 (saline) or 5 mg/kg KA. Two-way ANOVA revealed an effect of KA (p = 0.0008) but not of blade (p = 0.4838) nor an interaction (p = 0.7242; n = 4 per group). Posthoc tests revealed *p = 0.0157 for Sal-SP vs KA-IP blades, *p = 0.0245 for Sal-IP vs KA-IP blades. *I*: Number of seizures observed in the first 16 hours post injection with 5 mg/kg KA or 15 mg/kg KA (n = 4 for 5 mg/kg KA, n = 5 for 15 mg/kg KA; **p = 0.0079). *J*: Number of seizures exhibited in 2 hour bins following KA injection. *K*: Number of epileptic spikes/hr over the first 16 hours post injection with 5 mg/kg or 15 mg/kg KA (*p = 0.0159). *L*: Number of spikes observed in each 2-hr bin following KA injection. Dotted lines in *A*, *C*, *E* demarcate the boundaries of the GCL. Arrows indicate cFos-IR cells. Scale bar, 100μm.

Exploration of a novel, enriched environment reliably engages a sparse pattern of dentate granule cells, which can be assayed by the expression of the neuronal activity marker cFos [27–29]. We found a nearly two-fold increase in cFos-expressing dentate granule cells when mice were removed from their home cages and exposed to a novel, enriched environment (EE) for 2 hours, where interaction with new and unfamiliar objects stimulated the DG in a physiologic manner (Fig 1A and 1B).

When testing responses of the DG to varying doses of the excitotoxin kainic acid (KA), we discovered that treating mice with a low, non-seizure-inducing dose of KA (5 mg/kg) can sparsely activate neurons in the GCL, similar to physiologic stimulation. Interestingly, the effect of low-dose KA administration was not observed acutely, as it did not generate behavioral seizures (stage 1.7 ± 0.2 on a modified Racine scale; see Detailed Methods) or alter GCL expression of cFos within 2 hrs of administration (Fig 1C and 1D). However, 3 days after administration, 5 mg/kg KA increased the number of GCL neurons that expressed cFos by approximately two-fold that of saline-treated controls (Fig 1E and 1F). By contrast, a higher dose of KA (15 mg/kg) induced acute behavioral seizures (stage 3.6 ± 0.8; *p* = 0.04 vs. 5 mg/kg KA) that was associated with a robust increase in cFos expression 2 hrs after administration (Fig 1C and 1D), and decreased cFos expression 3 days after administration (Fig 1E and 1F), consistent with previous reports [26, 28, 30].

Previous studies have found differences in degree of neuronal activation, assessed by *Arc* or *zif268* expression, in the supra- and infrapyramidal blades of the dentate gyrus after environmental exploration, with the suprapyramidal blade exhibiting higher activity [31]. Indeed, when we examined whether the expression of cFos by dentate granule cells differed by blade, we found that the EE-induced increase was specific to the suprapyramidal blade, whereas the infrapyramidal blade did not exhibit increased cell activation (Fig 1G). Two-way ANOVA detected a significant effect of treatment (*p* < 0.0001), DG blade (*p* = 0.0013), and an interaction (*p* = 0.0003). Interestingly, although a low dose of KA 3 days after administration increased overall numbers of cFos-expressing granule cells to a similar extent as EE, the expression of cFos induced by KA did not vary by blade (Fig 1H). Two-way ANOVA detected a significant effect of KA treatment (*p* = 0.0008), but not of DG blade (p = 0.4838) nor an interaction (p = 0.7242).

To confirm that 5 mg/kg KA did not induce seizures that we could not detect behaviorally, we performed video-EEG recordings. One of four mice that received 5 mg/kg KA exhibited one electrographic seizure shortly after receiving KA, but no other seizures were observed afterwards, or in any other mouse, for three days post-KA injection (Fig 1I and 1J). In contrast, all mice that received 15 mg/kg KA exhibited seizures within the first 8 hours post-KA injection (Fig 1I and 1J). The total number of seizures observed within the first 16 hours after KA was significantly different between 5 mg/kg and 15 mg/kg KA (Fig 1I). We also plotted the number of seizures in 2-hr bins post-KA injection to gain perspective on the timing of seizures. Although two-way RM ANOVA did not detect a significant difference between these timelines, the graphs demonstrate the occurrence of a high number of seizures within the first 8 hours post injection of 15 mg/kg KA, but not for 5 mg/kg KA (Fig 1J). We also found that 5 mg/kg KA did not induce the occurrence of epileptic spikes, whereas 15 mg/kg KA did (Fig 1K and 1L). The number of spikes/hr in the first 16 hours post injection of 15 mg/kg KA was significantly higher than that of mice that received 5 mg/kg KA (Fig 1K). We also plotted the number of spikes observed in 2-hr bins post-KA injection (Fig 1L). Two-way RM ANOVA detect a significant effect of time (p = 0.025) and strong trends for effects of KA dose (p = 0.087) and interaction between timing and dose (p = 0.057). Together these results indicate that the low dose of 5 mg/kg KA does not generally induce seizures or epileptic spikes.

### Novel enriched environment exposure activates young and older dentate granule cells

We took advantage of the sparse activation of GCL neurons by 5 mg/kg KA to investigate how the DG responded to non-physiologic stimulation comparable in magnitude to physiologic stimulation. As mentioned previously, intrinsic properties of the DG network (e.g., neurogenesis and GABAergic tone) are thought to contribute to the sparse activation of neurons in the GCL when the DG is stimulated. However, whether the etiology of DG stimulation (physiologic versus pharmacologic) plays a major role in the selective engagement of specific neuronal types is unknown. Presumably, if intrinsic properties of the DG circuitry are sufficient to generate a sparse and selective pattern of cell activation in the GCL, then stimulation of the DG via 5 mg/kg KA should activate cells in the same manner and pattern as EE. Therefore, to assess whether the sparse and selective activation of GCL neurons was a unique property of physiologic stimulation, or could be replicated by a low-intensity, non-physiologic stimulus, we compared the repertoire of cell types in the DG that were activated by physiologic (EE) or low-level aberrant (5 mg/kg KA) stimulation.

**Fig 2:**
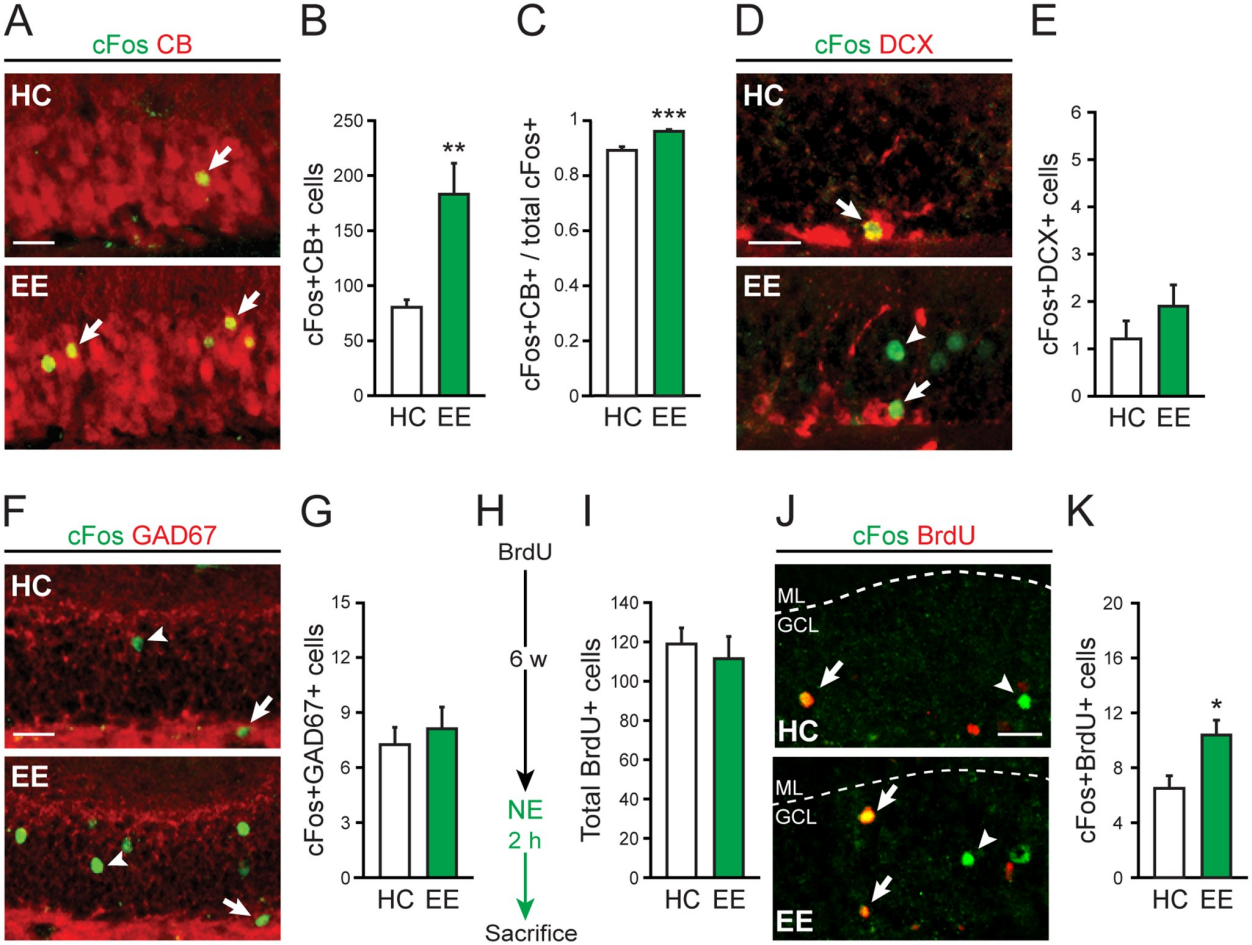
EE activates young granule cells. *A*,*B*: Representative images and quantification of calbindin (CB)-positive cells that express cFos in the GCL after EE exposure versus home cage (HC) control (n = 8 per group; **p = 0.0032). *C*: The proportion of cFos-expressing cells that are CB-positive after EE exposure (n = 8 per group; ***p = 0.00027). *D*,*E*: Images and quantification of doublecortin (DCX)-positive cells that express cFos in the GCL after EE exposure (n = 10 per group; *p* = 0.26). *F*,*G*: Images and quantification of GAD67-positive cells that express cFos in the GCL after EE exposure (n = 8 per group; *p* = 0.57). *H*: Experimental timeline for BrdU injection and EE exposure. *I*: Quantification of BrdU-labeled cells in the GCL in mice exposed to EE 6 weeks after BrdU injection (n = 8 per group; *p* = 0.6). *J*,*K*: Images and quantification of cFos and BrdU co-labeling in the GCL after EE exposure (n = 8 per group; *p = 0.017). In panel *J*, dotted lines demarcate the boundaries of the GCL. Arrows indicate cells that express both cFos and CB (panel *A*), cFos and DCX (panel *D*), cFos and GAD67 (panel *F*), or cFos and BrdU (panel *J*). Arrowheads indicate cells that only express cFos in corresponding images. Scale bar, 25μm.

We first classified the types of cells in the GCL that were activated to express cFos in wildtype mice exposed to EE. We found that nearly all of the cells activated by EE were more mature granule cells (defined here as ≥4 weeks of age) (Fig 2A and 2B), since they expressed calbindin [32, 33]. This finding was not surprising, since granule cells are the predominant cell type in the GCL [34], and previous studies have demonstrated that their synaptic responses stabilize at ~4 weeks of age [7, 35]. Notably, we found that the proportion of cFos-expressing cells that also express calbindin was increased by EE exposure (Fig 2C), indicating that physiologic stimulation of the DG preferentially activated the more mature granule cells over other GCL cell types. Indeed, EE exposure did not induce cFos expression in immature granule cells < 4 weeks old (Fig 2D and 2E), which express doublecortin [33], or GABAergic interneurons (Fig 2F and 2G), which express GAD67 [36].

We then asked whether EE exposure activated mature granule cells of various ages including young, recently matured granule cells (6–8 weeks of age). To answer this question, we labeled a cohort of newborn neurons in the GCL by treating naïve mice with BrdU, a cell division marker that is incorporated into dividing cells (such as neural progenitors in the GCL) and is passed on to their progeny [37]. We waited 6 weeks for the labeled neurons to develop and mature, and then exposed BrdU-treated mice to EE. After EE, mice were sacrificed and their brains processed to assess co-labeling of cFos and BrdU (Fig 2H). We found that overall uptake of BrdU by the cells in the GCL was equivalent between the mice that were exposed to EE versus home cage controls (Fig 2I). However, EE exposure increased the number of BrdU/cFos co-labeled cells (Fig 2J and 2K), indicating the activation of younger granule cells by physiologic DG stimulation.

### Low-dose kainic acid activates older dentate granule cells and interneurons

**Fig 3:**
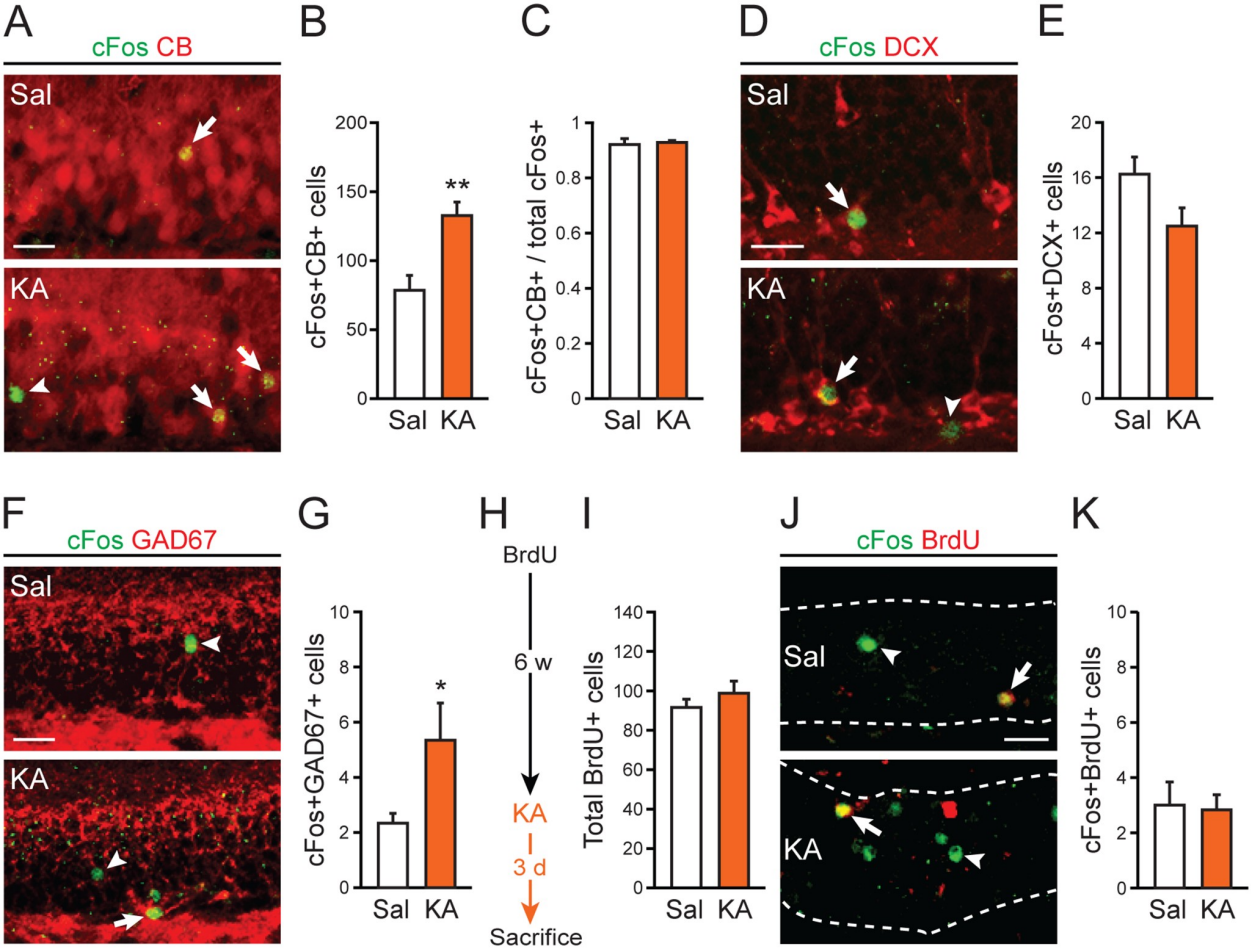
Low-dose KA activates older dentate granule cells and GABAergic interneurons. *A*,*B*: Representative images and quantification of calbindin (CB)-positive cells that express cFos in the GCL 3 days after 5 mg/kg KA administration versus saline (Sal) control (n = 4 per group; **p = 0.0098). *C*: The proportion of cFos-expressing cells that are CB-positive after 5 mg/kg KA administration (n = 4 per group; *p* = 0.75). *D*,*E*: Images and quantification of doublecortin (DCX)-positive cells that express cFos in the GCL after 5 mg/kg KA administration (n = 4 per group; *p* = 0.09). *F*,*G*: Images and quantification of GAD67-positive cells that express cFos in the GCL after 5 mg/kg KA administration (n = 10–12 per group; *p = 0.032). *H*: Experimental timeline for BrdU injection and 5 mg/kg KA administration. *I*: Quantification of BrdU-labeled cells in the GCL in mice treated with 5mg/kg KA 6 weeks after BrdU injection (n = 6–8 per group; *p* = 0.32). *J*,*K*: Images and quantification of cFos and BrdU co-labeling in the GCL after 5 mg/kg administration (n = 6–8 per group; *p* = 0.88). In panel *J*, dotted lines demarcate the boundaries of the GCL. Arrows indicate cells that express both cFos and CB (panel *A*), cFos and DCX (panel *D*), cFos and GAD67 (panel *F*), or cFos and BrdU (panel *J*). Arrowheads indicate cells that only express cFos in corresponding images. Scale bar, 25μm.

We next evaluated how 5 mg/kg KA affected cFos expression in various GCL cell types 3 days after administration. Similar to EE, the majority of cells activated by 5 mg/kg KA were calbindin-positive, more mature granule cells (Fig 3A and 3B). However, in contrast to EE stimulation, 5 mg/kg KA treatment did not increase the proportion of cFos-expressing cells that also express calbindin (Fig 3C), suggesting that other types of neurons within the granule cell layer were also activated. We found that 5 mg/kg KA did not affect cFos expression in immature, doublecortin-expressing granule cells (Fig 3D and 3E), but did increase cFos levels in GAD67-expressing interneurons (Fig 3F and 3G), indicating increased activity of GABAergic interneurons. Lastly, we treated a separate cohort of naïve mice with BrdU, and 6 weeks later assessed whether 5 mg/kg KA also affected the number of cells that co-labeled with both cFos and BrdU (Fig 3H). We found that uptake of BrdU by the GCL was equivalent between BrdU-treated mice that were later treated with saline or with 5 mg/kg KA (Fig 3I). However, unlike EE, 5 mg/kg KA treatment did not increase the number of BrdU/cFos co-labeled cells (Fig 3J and 3K), suggesting that 5 mg/kg KA did not activate younger granule cells.

### Novel enriched environment and low dose kainic acid activate different patterns of DG cells

**Fig 4:**
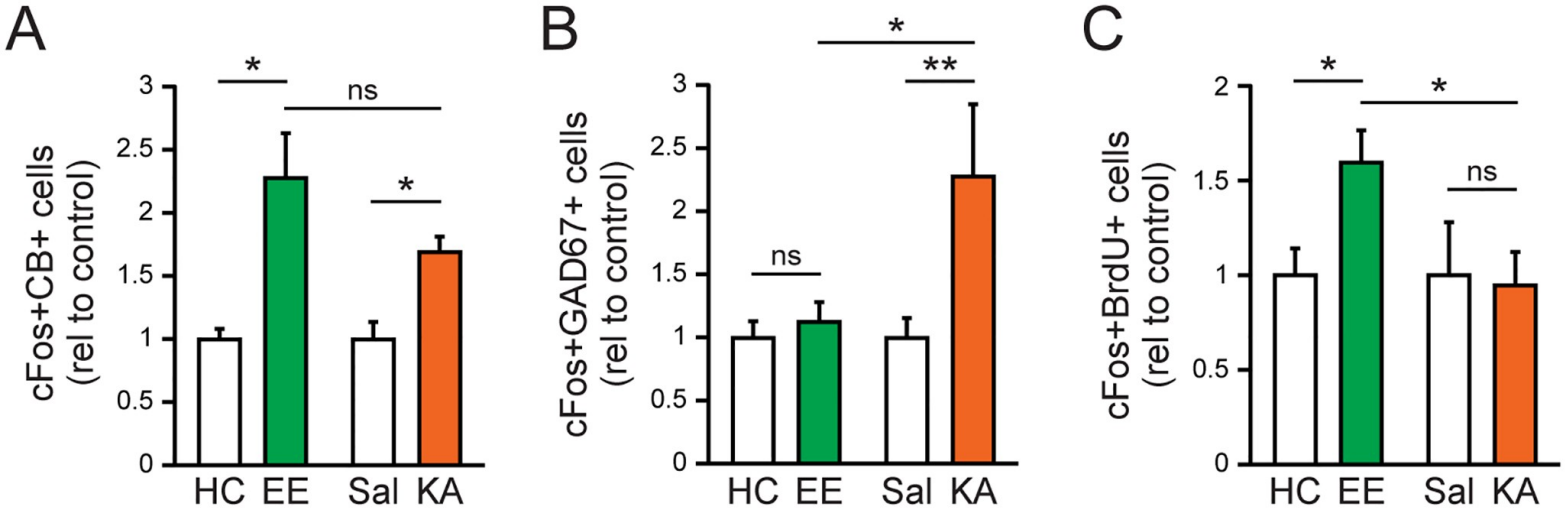
Enriched environment and low-dose kainic acid activate different patterns of cells in the GCL of the DG. *A*: Direct comparison of cFos and CB co-labeling between mice that were exposed to EE and those that received 5 mg/kg KA (n = 4–8 per group; One-way ANOVA followed by post hoc tests: EE vs. HC, *p = 0.04; KA vs. Sal, *p = 0.048; EE vs. KA, *p* = 0.55). *B*: Direct comparison of cFos and GAD67 co-labeling between mice that were exposed to EE and those that received 5 mg/kg KA (n = 8–12 per group; One-way ANOVA followed by post hoc tests: EE vs. HC, *p* = 0.81; KA vs. Sal, **p = 0.007; EE vs. KA, *p = 0.025). *C*: Direct comparison of cFos and BrdU co-labeling between mice that were exposed to EE and those that received 5 mg/kg KA (n = 6–8 per group; One-way ANOVA followed by post hoc tests: EE vs. HC, *p = 0.043; KA vs. Sal, *p* = 0.86; EE vs. KA, *p = 0.041). ns, non-significant. Cell count values for EE and KA have been normalized to HC and Sal controls, respectively.

The experiments above that examined the effects of EE and 5 mg/kg KA kainic acid were performed and assessed independently. To directly compare whether the patterns of DG cell activation were different between the two paradigms, we analyzed the data across all experimental conditions after normalizing the cell counts of the experimental condition (e.g., EE or 5 mg/kg KA kainic acid) to their respective controls (e.g., home cage or saline). Consistent with our independent analyses, we found increased co-labeling of cFos and calbindin in the GCL of mice exposed to EE as well as those that received 5 mg/kg KA kainic acid, indicating that the two stimulation paradigms induced cFos expression in more mature, calbindin-expressing granule cells (Fig 4A). However, only 5 mg/kg KA kainic acid induced cFos expression in GABAergic interneurons, since there were significantly more cFos/GAD67 co-labeled cells in mice that received 5 mg/kg KA compared to saline-treated mice or mice that were exposed to EE (Fig 4B). Finally, we found that EE exposure significantly increased co-labeling of BrdU and cFos in the GCL, an effect that was not observed with 5 mg/kg KA administration (Fig 4C). This result demonstrates that EE activated a greater number of younger granule cells compared to 5 mg/kg KA administration.

## Discussion

Our study highlighted a notable difference between the responses of the DG to physiologic stimulation (EE) and low-level pharmacologic stimulation (5 mg/kg KA). Despite activating a similarly sparse number of cells in the GCL, 5 mg/kg KA did not mimic the specificity of cell types engaged by EE. EE activated younger (6–8 weeks old) and older (≥ 8 weeks) granule cells, whereas 5 mg/kg KA appeared to have activated older granule cells (≥ 8 weeks) as well as GABAergic interneurons, but not younger granule cells (6–8 weeks old). The engagement of older granule cells by low-dose KA may provide an explanation for the simultaneous increase in cFos and GAD67 co-labeling in KA-treated mice, since activation of older granule cells largely drives inhibitory feedback via reciprocal connections with GABAergic interneurons [38, 39]. Overall, this study indicates that the specific pattern of cell activation important for memory encoding is not simply a function of stimulation intensity but is influenced by a complex interplay of factors unique to the physiologic stimulus. Moreover, the intrinsic properties of the DG network may be necessary but are not sufficient for generating the sparse and particular pattern of GCL activation during physiologic stimulation; factors external to the DG likely play important roles as well.

Such factors could include the etiology of DG stimulation. A previous study demonstrated that high frequency stimulation of the perforant path, which carries information from the entorhinal cortex to the DG, elicited cFos expression in nearly 100% of mature, calbindin-expressing dentate granule cells [40]. Similar to our findings, the authors in the previous study found that very few DCX-expressing immature neurons in the DG expressed cFos upon stimulation. However, using a combination of BrdU labeling and perforant path stimulation, they found that 75% of DG cells that were 35–77 days old could be activated. This finding indicates that activation of the DG via stimulation of endogenous afferents can activate and induce cFos expression in both younger (<8 weeks) and older (≥ 8 weeks) mature granule cells, which provides an interesting contrast to our findings that nonspecific stimulation of glutamate receptors with KA activated only older granule cells along with interneurons. These results indicate that activation of the DG via stimulation of endogenous afferents engages a more physiologically relevant set of DG cells, compared to nonspecific pharmacological activation of the DG. These results also suggest that pharmacological methods that enhance glutamate receptor signaling to boost neuronal activity [41–43] may benefit from characterization of cell types that are activated.

In summary, our study compared physiologic stimulation of the DG (exploration of a novel enriched environment) to aberrant pharmacological stimulation of the DG (low-dose KA). Both methods of stimulation generated similarly sparse activation of DG cells, but with differing patterns of cell types. These findings indicate that local circuit and network properties shape the pattern of DG activation when stimulation occurs via physiologic afferent routes, but that these properties can also be over-ridden by aberrant forms of stimulation.
